# Positive Relational Experiences in Infancy May Influence Outcomes in Children in a Low and Middle-Income Country Setting Such as South Africa

**DOI:** 10.3389/fpubh.2021.665908

**Published:** 2021-08-18

**Authors:** Astrid Berg, Anusha Lachman

**Affiliations:** Department of Psychiatry, Stellenbosch University, Cape Town, South Africa

**Keywords:** relational experiences, infant mental health, LMIC setting, infant mental health screening, parenting intervention

## Introduction

The infant represents hope and a new beginning. Generally speaking, most parents and those around the baby are intent on doing all they can to facilitate a good start to life. When things go awry, either physically or emotionally, every effort is usually made to enable a return to a positive path. This effort may be made by the parents themselves, in that they will take on the responsibility of nurturing care instinctively, even if it requires effort and sacrifice. Other parents or caregivers may need help from community based workers and even specialized units. Whatever approach may be used, it will almost always involve the infant and his/her caregivers or parents and will focus on the relationship between them.

Daniel Stern has outlined the unique aspects of therapeutic approaches which aim to help the parent-infant relationship (1). Two of the eight points he makes are worth highlighting in the context of this article: That the infant has an “infant psyche [that is] neither neurotic, borderline or psychotic” (p. 3)—with other words, there is no pathology in the infant psyche. Secondly, that the parents are “psychologically ‘normal,’ in the vast majority of cases” (p. 3).

It thus follows that in the field of infant mental health the emphasis lies in supporting health, rather than seeking out pathology. As such, it falls within the ambit of positive psychology, a concept explored by Seligman in 2000. It is “an umbrella term for the study of positive emotions, positive character traits, and enabling institutions” (2) (p. 410). The hallmarks of positive psychology are, amongst others, the fact that positive emotions are widely recognized across cultures, and that they are seen to be contributors to individual fulfillment and satisfaction and that the focus should be on these, rather than on psychopathology.

## The Role of Positive Emotions in Parent-Infant Interaction

Positive emotions expressed in the parent-infant relationship are not only enjoyable to witness, but play a crucial role in the development of the brain (3). Situations that offer positive responses are important for the maturation of the behavioral approach system (4). What is now being supported by neuroscience, was intuited many years ago by clinicians working in the field.

Positive emotions serve to strengthen the relationship between caregiver and infant and in turn a good enough early relationship will bode well for the child's future emotional development. Positive emotions shared in meaningful relationships build social, intellectual and psychological resources for the infant throughout their life span (5). It is the investment in the early years that gives the highest returns in terms of improved physical and mental health (6). Selma Fraiberg was the pioneer in parent-infant psychotherapy in the USA. She dared to move beyond the confines of the psychoanalytic setting and visited families in their homes (7).

She describes the enormous responsibility that lay with the clinician when it was known that there were “two patients behind the door, and that one of them is a baby” (p. 408). One of the cases she described in detail is that of baby Greg. Although everything in this family seemed bleak and hopeless, there emerged moments of hope. After talking about her own childhood traumas, Annie, the young mother, was able to spontaneously pick up Greg and cuddle him. These “small positive signs” (p. 405) are the “moments of meeting” that can catalyse change in the parent-infant relationship (8) (p. 286).

The global situation for infant-mental health has shifted dramatically since the times of Selma Fraiberg. The intensive parent-infant psychotherapy that she and her team were able to offer is often not do-able, particularly so for resource constrained settings. In addition, such in-depth work that was offered to Annie and the other parents in their project may not be indicated, nor may it be necessary for a broad section of the population. However, the basic principle of looking out for “small positive signs” and for facilitating “moments of meeting” remain central to most intervention programmes.

## Early Screening and Intervention in South Africa

In a LMIC setting, such as South Africa, it is of importance to recognize relational difficulties between infant and caregiver early, and to be able to offer evidence-based interventions that are brief and focused on strengthening the relationship. Two different modalities are described here, one a screening for the very early identification of a possible relational “alarm” signal—-the absence of reciprocal synchronized shared pleasure—the other a reflective parenting intervention that has been shown to improve maternal sensitivity and reflective functioning. Both of these have and continue to be incorporated into local primary prevention programmes.

## The Basic Infant Mental Health Screen

Based on findings in earlier studies, the Basic Infant Mental Health Screen (BIMHS) was developed in collaboration with colleagues from Finland (9). This is a simple and short tool which focuses on infant mental health. The screening is meant to be routinely done by any therapists or allied health care professionals who assess infants in different settings of care. These settings which do not routinely assess mental health may include social evaluations, feeding assessments, well-baby clinic or immunization visits for the infant. If any concerns are raised the health care worker is advised to follow up with the dyad at the next visit and repeat the BIMHS or to refer on to mental health clinics. Among the 5 items is one of particular interest, and that is the component of noting Shared Pleasure (SP) between infant and caregiver.

SP in parent-infant interaction is defined as “the parent and the child sharing positive affects in synchrony” (10). It is hypothesized that the sharing of a smile together with direct eye contact between a mother and an infant is a marker of high intensity positive affect. Most compelling in the use of SP as a screening marker is the potential that a shared positive affect between mother and infant may correlate with attachment security, and may be highly malleable in the first 12 months of life (11). In a recent study looking at contributors to SP occurrences, dyads where mothers and infants were best able to read each other's positive cues and respond to them were more likely to experience a SP. Additionally, lower depressive symptoms, greater infant involvement and better maternal structuring of the interaction were associated with a longer duration of SP (12). Currently the measurement of SP is done by direct observation by the therapist or health care provider with the mother and infant in the room. If there is a sense of there being a reduced reciprocal pleasurable interaction, reduced eye contact or lack of joyful engagement, the therapist is encouraged to review the interaction at a different time and in the context of the other BIMHS screening questions. SP assessment is a new concept in the South African setting, and the roll of out of the BIMHS with routine infant medical stationary alongside research initiatives with the SP paradigm is ongoing. How well clinicians are able to identify SP and if this improves early referrals remains to be seen. SP may hold potential for a simple observational measure of synchrony in the mother-infant relationship and its absence could be considered a flag of risk in the dyad.

## Mothering From the Inside Out

Mothering from the Inside Out (MIO) is an evidence based intervention, developed in the USA by Nancy Suchman, for mothers of toddlers who were experiencing severe psycho-social stressors. In a mixed-method study, the feasibility and acceptability of adapting this parenting intervention was examined in 5 public health settings in the Western Cape (13). Vulnerable mothers of young children were seen for 8–12 weekly sessions, either individually or in groups, depending on what was most acceptable to a particular setting. Pre- and post-intervention measures were done.

The first principle of the intervention was on establishing a “frame,” that is consistency in time, place and ensuring confidentiality. The second was providing a space for sharing difficult moments with their children. It was the therapist's task to contain the painful feelings and to gradually get the mother to a point where she could think about what happened, reflect on her own feelings and how that affected her behavior, and how this in turn affected her child. It was the process of consistency, containment, and being able to step back, slow down and reflect that were the essential components of this process.

One of the health settings exploring the intervention was a Kangaroo Mother Care (KMC) Facility at a tertiary hospital in Cape Town. KMC or “skin to skin” contact is considered optimal care for low-birth-weight preterm infants in developing countries. It is considered a cost efficient and effective alternative to neonatal ICU care. Mothers find themselves in KMC units mostly for reasons that are unpredictable and unexpected as can be anticipated in preterm births. Following a preterm birth the mother-baby relationship is more difficult as interactions are complicated by babies with reduced expressions and responsiveness, and mothers with lower sensitivity. Maternal fragility often leads to lower levels of synchronicity. With this in mind, the MIO intervention was adapted into a group setting at the KMC unit. Groups were structured as a supportive psychodynamic intervention focused on providing a safe space with positive input, and to support mothers developing the capacity to mentalize and reflect about their own and their babies emotional experiences.

With limited capacity to tolerate or make sense of internal affective states (their own or their young children's), the mother often faces unbearable and frightening distress. We used a curious and questioning approach to mothers through reflection and open-ended questions. We noticed over time that many mothers did not have a safe therapeutic space in which to share their conflicting and ambivalent feelings, having been forced unprepared into an unfamiliar environment in the first few days of their babies lives.

Therapists adopted a respectful approach holding a stance of curiosity and willingness to learn from the mothers. Not surprisingly, a positive therapeutic alliance cast the therapist into a role of a “good enough parent.” What unfolded initially was the security to share the guilt, frustration and helplessness of the setting, without judgement. This was followed by an unexpected openness and curiosity from mothers. Delight in noticing the small attempts by the baby to connect once attention was drawn to them by the therapists. Mothers looked forward to the small groups where they could enjoy the progress in little ways—being encouraged to find the joy in the babies preferential responses to their mothers' voices or babies' comfort and soothing from the proximity of the mothers' skin and heart rate. To quote a participant—“it was new and nice to notice my baby in a positive way—not just as a too small baby who isn't drinking or growing enough.”

## Conclusion

The focus on positive relational experiences is a characteristic of infant mental health interventions. The infant's need for affirmative relationships is immediate and urgent, and if absent, needs to be flagged as a potential indicator of early difficulties. Universal screening of infants during routine vaccination procedures provides an opportunity to observe the interaction between caregiver and infant. If health care providers can be made aware of the importance of positive engagement, they may be able to follow up those dyads where this is absent and intervene if necessary.

Similarly, giving vulnerable mothers of premature babies the opportunity to share anxieties and fears, will open up the possibility of noticing “small positive signs” of their tiny infants, the importance of which cannot be overestimated. At the beginning of life every positive experience, no matter how small to our adult eyes, can help to lay the foundation for future mental and physical health.

## Author Contributions

AB and AL contributed to the conception and design of the manuscript. AB wrote the first draft. AL wrote sections of the manuscript. Both authors contributed to the manuscript revision, read, and approved the submitted version.

## Conflict of Interest

The authors declare that the research was conducted in the absence of any commercial or financial relationships that could be construed as a potential conflict of interest.

## Publisher's Note

All claims expressed in this article are solely those of the authors and do not necessarily represent those of their affiliated organizations, or those of the publisher, the editors and the reviewers. Any product that may be evaluated in this article, or claim that may be made by its manufacturer, is not guaranteed or endorsed by the publisher.
